# Architecture to Embed Software Agents in Resource Constrained Internet of Things Devices

**DOI:** 10.3390/s19010100

**Published:** 2018-12-29

**Authors:** Daniel H. De La Iglesia, Gabriel Villarrubia González, André Sales Mendes, Diego M. Jiménez-Bravo, Alberto L. Barriuso

**Affiliations:** Computer and Automation Department, University of Salamanca, Plaza de la Merced s/n, 37008 Salamanca, Spain; gvg@usal.es (G.V.G.); andremendes@usal.es (A.S.M.); dmjimenez@usal.es (D.M.J.-B.); albarriuso@usal.es (A.L.B.)

**Keywords:** software agents, Internet of Things, WSN, smart devices

## Abstract

Sensing systems in combination with treatment tools and intelligent information management are the basis on which the cities and urban environments of the future will be built. Progress in the research and development of these new and intelligent scenarios is essential to achieve the objectives of efficiency, integration, sustainability, and quality of life for people who live in cities. To achieve these objectives, it is essential to investigate the development of cheaper, more accurate, and smarter hardware devices, which will form the basis of the intelligent environments of the future. This article focuses on an approach based on intelligent multi-agent systems that are integrated into basic hardware devices for the Internet of Things (IoT). A multi-agent architecture is proposed for the fast, efficient, and intelligent management of the generated data. A layer of services adapted to the needs of the new intelligent environments is built on this architecture. With the aim of validating this architecture, a case study based on electric vehicles of the e-bike type is proposed.

## 1. Introduction

At present, citizens live in a global world where people and objects are increasingly interconnected, regardless of their geographical location. Buildings, cities, vehicles, Smartphones, and other devices are already equipped with digital sensors capable of recording large amounts of data [1]. Sensing systems represent a fundamental element in the actual information age. Thanks to these systems, it is now possible to measure certain values that previously could not be measured. These new data have been a revolution in the way in which users interact with the environment. To a large extent, this advance has been motivated by the price of sensors, which has been decreasing during the last ten years. Estimations indicate that sensor manufacturing and design processes will be reduced by approximately 5% per year in the next decade [2]. This prolonged drop price will mean significant savings when installing large-scale sensors in different environments, such as those needed to connect a large number of devices in an IoT (Internet of Things) environment [3]. 

The IoT concept is one of the most used in recent years to refer to all those connected devices that make up intelligent environments. One of the main problems that IoT environments face is the amount of data that their devices collect and with which, in many cases, nothing is done. These data are usually sent to central servers in the cloud, where they are analyzed to obtain derived information or activate certain events. For this reason, concepts such as Edge Computing [4] or Fog Computing [5,6] have emerged, which seek to change this passive behavior of the IoT nodes and turn devices into an active part of the system. By providing these devices with data analysis mechanisms, processing of the data closest to the place where they are created is achieved instead of being sent through long routes to reach external data centers.

Bringing data processing to local environments makes even more sense if growth forecasts for the next few years are analyzed in the number of devices connected to the Internet that generate data. According to the report [7], it is expected that by the year 2020, the number of connected devices will surpass 50 billion, which will mean that for each citizen, there will be almost seven connected devices emitting data. Therefore, it is important that these data are processed in the devices themselves to avoid network saturation, unnecessary redundancy, and higher data storage costs. This local processing will improve the efficiency by not having to transmit all these data to the cloud, assuming significant cost savings and an increasement in processing speed. For certain sectors such as industry, health, transportation, telecommunications, or finance, among others, the real-time analysis of data is a fundamental need that is achieved with a faster processing of recorded data.

One of the main problems currently existing is the lack of intelligence implemented on the deployed sensor devices. To a large extent, this lack is due to the limited computational resources (memory, computing capacity, and electrical consumption) of these devices. Even though the industry is getting closer and closer to developing lightweight, efficient devices with high capacities, the reality is that, at this moment, the majority of available sensors and actuators are limited computationally. In order to achieve a real revolution from the analog world to a digital world with intelligent environments, it is necessary to incorporate intelligence capabilities and mechanisms into existing devices on a day-to-day basis. By achieving this objective, intelligent and technologically and economically viable environments will be achieved.

This paper proposes an integral IoT framework based on multi-agent systems for the intelligent management of computationally limited hardware devices that are the basis of the deployed sensor systems. The proposed system seeks, on the one hand, to provide IoT devices with intelligence in order to achieve decentralized, prior data processing with a greater efficiency. On the other hand, it seeks to provide a platform based on intelligent software agents able to efficiently manage all the information derived from the IoT systems on which to develop services and products that benefit citizens. The way in which this intelligence is achieved is through embedding a reactive agent in the hardware devices. This agent will be part of the MA (multi-agent) architecture as one more agent, regardless of its location, type, or characteristics. Thanks to this abstraction, the system will be able to execute artificial intelligence mechanisms on the data and processes generated by the agent embedded in the device. It is not therefore a simple input/output agent, and this agent (through its abstraction in the system) will take an active role in the management of data and processes carried out in the main system.

The work carried out seeks to provide a solution to the problems posed by the authors in [8], where a series of needs and requirements are introduced when building intelligent IoT environments that are adapted to the changing circumstances.

This article is structured as follows: Section 2 reviews the current state of the art work; Section 3 describes the proposed system in detail; Section 4 presents the case study which was conducted to validate the proposed architecture and the results obtained. Finally, conclusions drawn from the work are outlined in Section 5. 

## 2. Background

In the current technological framework, the development, management, and integration in real environments of IoT systems, such as Smart Homes or Smart Factories, represent a complex challenge for all the agents involved in its development and exploitation. That is the main reason why it is necessary to design and apply appropriate models, methodologies, technologies, and paradigms that help developers to address these new challenges. In this sense, different solutions, tools, and middleware methodologies have been developed that aim to address these problems. As the authors of [9,10] explain, it is possible to group solutions based on their design approaches, resulting in different groups such as service-based architectures [11], event-based architectures [11], or architectures based on agents [12]. 

The latter are those based on software agents, which are addressed in this work. Specifically, an analysis of the current state of the art has been carried out on platforms responsible for providing intelligence to the computationally limited devices that make up the current IoT. For the authors of [13], the solution is to implement mobile agents on hardware devices. Specifically, this work is committed to work with hardware devices that can run TinyOS, an open source operating system specially designed for wireless sensor networks. This operating system is specially designed for a series of platforms (Telos, Mica2Dot, Mica2, Mica) so it is not possible to integrate it into other devices than those. In the case of [14], the authors also bet on the integration of mobile agents as a method to provide intelligence to the hardware nodes. The authors highlight the fact that their implementation, as suggested by the work of [13], can also be displayed on TinyOS or on a Java MV. Specifically, it can also be displayed on Sun SPOT devices, compatible with Java programming, but with a high price, so this has been discontinued.

In contrast, in [15], the authors propose the integration of a mobile agent with the rest of the architecture through the implementation of a REST API. This API provides the creation and control of mobile agents, the migration of agents, the communication between devices and agents, and the local and remote access to available resources. It is therefore a new approach, where the exchange of data between the nodes and the rest of the platform through an API is the key to a functional system. In previous research [16], the authors made use of Self-StarMAS [17], a lightweight implementation based on the architecture of Malaca agents [18]. It is an architecture in which the agent is specially designed, taking into account the restrictions of the resources and capabilities of each device (for example, the communication protocol used). The different versions of Self-StarMAS can be run on Android devices, mobile phones with an MIDP profile (Mobile Information Device Profile), desktop computers, Sun SPOT sensors, and Libellium Waspmotes. The main characteristic of the internal architecture of a Self-StarMAS agent is that it is composed of a set of components that help to keep the specific functionalities of the application of the concrete communication protocols uncoupled. Therefore, through Self-StarMAS, it is possible to integrate new important devices, such as Android terminals or Waspmotes sensors.

The concept of a software agent for the authors of [19] is used theoretically and no methodologies, implementations, or specific techniques are described. As previously commented, the work of the authors [20], despite being one of the most complete available in the literature, does not delve into the methods or designs employed to embed software agents.

As a conclusion and after having analyzed the most relevant works of recent years in the field of study of this work, it is possible to identify some notable shortcomings. In recent years, there have been numerous works that address the main issues of the management of IoT environments through multi-agent systems and software agents embedded in limited hardware devices. It has not been possible to find works that delve into mechanisms and design concrete solutions to carry out these developments. It is also possible to identify a lack of specific implementations and developments, deepening and detailing the code and programming languages used. Another aspect that stands out is the lack of concrete security and confidence mechanisms of the MAS systems proposed and described by the authors of the different works analyzed. 

Having considered the points above, this work seeks to provide a concrete solution, and proposes a concrete and well-defined software agent model to embed software agents in computationally limited devices, as well as security and trust mechanisms appropriate to the needs raised in current IoT environments. 

## 3. Proposed System

As discussed in the previous section, this work seeks to provide solutions to the challenges posed by the authors of [8] when addressing the design of dynamic IoT environments through the incorporation of software agents on computationally limited wireless hardware devices. In the work of the authors, two fundamental concepts are introduced when modeling IoT environments: Smart Objects (SOs) and Smart Environments (SEs). So-called SOs are the fundamental components of IoT environments and refer to all those everyday objects that are equipped with hardware components such as a processor, a communication system, and sensors or actuators to take control of the environment, measuring and acting on it [21]. On the other hand, the concept of SE is defined as a cyber-physical environment where different heterogeneous devices are used, capable of collecting data from the environment and interacting with the environment [22,23].

The main requirements posed by the authors of [8] and to which an answer is proposed are detailed in Table 1.

### 3.1. Limited Hardware Devices for the IoT

In the IoT, light hardware devices that can be integrated into a multitude of ‘things’ and which form the basis of the cyber-physical systems involved in the Internet of Things, play a fundamental role. It is necessary, therefore, to define what is meant by computationally limited devices or limited resource devices. 

**Figure 1:**
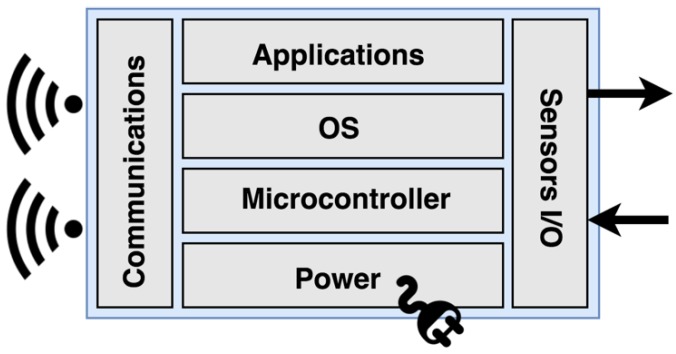
Basic architecture of an IoT device.

In general terms, these devices may be limited by their size, by their data storage, by their energy source, by their computing capacity, or by their communication capacity. Therefore, the tasks carried out by these devices are usually highly optimized. The main elements (software and hardware) present in an IoT device are described in Figure 1.

Currently, there are different hardware devices on the market with reduced prices that can be incorporated into a large number of ‘things’ and that can expand the horizons of the IoT, as well as help students and educators to enter the world of cyberphysical systems. There are numerous developments, kits, and boards from different manufacturers, but there are two that stand out from the rest of them at present: ESP8266 and ESP32.

This is the case for ESP8266 modules from the Espressif company, which have a complete TCP/IP stack together with a microcontroller, which allows them to connect to WiFi networks in a simple way, as well as incorporate external sensors and actuators, all with a very low cost of just one euro. The ESP8266 module is therefore a more complete solution for IoT projects. Arduino devices are used for simple IoT projects or prototypes, where the inclusion of these boards is performed with an ad hoc topology. On the contrary, and due to their characteristics, ESP8266 modules can be easily integrated into new industrial developments, based on IoT. Therefore, many of the devices that are currently marketed on the IoT market have this module incorporated. In addition to using the C and C++ languages, the ESP8266 module can be programmed in Lua or JavaScript, while the Arduino board can only be programmed in the C language. Thanks to the success of this module, a series of devices have emerged in recent months. The prototyping and development of IoT projects based on the ESP8266 has resulted in the NodeMCU or WeMos. The ESP32 module is the evolution of ESP8266 and is more focused on the evolution of the IoT. The ESP32 is a much more powerful device, and uses an Xtensa dual-core processor LX6 32-bit 160 or 240 MHz. It has two processing cores, which allows one of them to be dedicated to communication and the other to the remaining processes, which is an advance with respect to the ESP8266 module. The main disadvantage of this module is its cost increase (around 6 euros) with respect to ESP8266. The operation of ESP8266 and ESP32 is very similar, and they are two microcontroller devices that can be programmed using the USB-to-TTL communication interface. There are several ways to program these devices to make them work in IoT environments. The most practical and simple method is to program them through the Arduino IDE. These boards have a series of GPIO (General Purpose Input/Output) pins that can be used as data entry and exit lines. In this way, and counting on these entry lines, these devices work by analyzing the data of the environment and reacting to them.

As it has been proven, both the ESP8266 and ESP32 boards are very focused on the development of IoT systems, are widely used, and have become an industry standard. Therefore, this work will focus on the development of these IoT devices. 

### 3.2. Agent and Multi-Agent Concept

**Figure 2:**
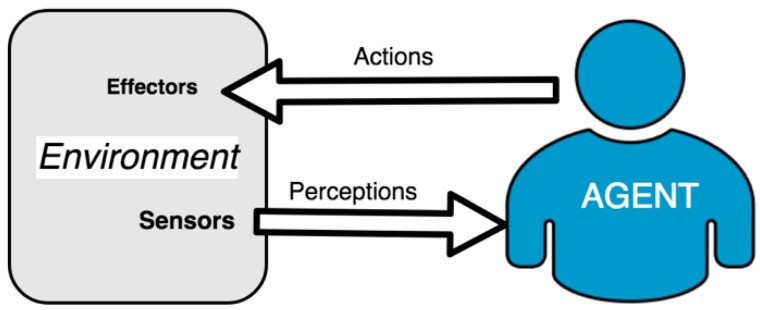
Agent model.

Smart software agents are a series of entities that seek to emulate human reasoning processes or behaviors. It is possible to find agents focused on helping users, performing certain tasks, performing supervisory and security tasks, or filtering information, among others. Based on these definitions, Figure 2 shows the basic diagram of the relationship between an agent and the environment in which it is deployed. It is possible to observe how the agent perceives its environment through a series of sensors and subsequently intervenes in it through effectors.

It is necessary to highlight the difference between a multi-agent system and an agent-based system. The systems based on agents are those systems that work with the concept of agent as an abstraction mechanism when modeling a system, but when implementing it, this can be done through traditional software structures. In contrast, a multi-agent system is designed with the clear idea that it will be composed of several agents that interact with each other in order to achieve a desired functionality [24].

When a problem exceeds the capabilities of an individual agent, several agents can be integrated into an MAS (Multi-Agent System) that provides a framework for agents to communicate and cooperate in solving problems [25]. An MAS is characterized as a system where each agent has incomplete information or capabilities to solve a given problem, it has no global control, it has decentralized data, and its tasks are executed asynchronously. Each individual agent, within an MAS, has its own objectives and knowledge, so coordination is required to combine these contributions and solve the problem addressed [26]. An MAS is generally considered a robust system since the failure of a single agent does not endanger the operation of the entire system. The agents in an MAS are able to learn and plan actions in a cooperative way.

### 3.3. Architecture Overview

When dealing with the integration of software agents on wireless devices, it is necessary to establish a clear differentiation between the types of connection available on these devices. A valid classification is that offered by the authors of [27] and defined as ‘classification based on their network stacks’. In this classification, the level of integration between the Internet and wireless devices depends on the protocols and network interfaces they implement. A device can be completely independent from the Internet (Front-End), be able to exchange information through an intermediate host (Gateway), or use a complete network protocol such as TCP/IP. For this work, the devices framed in the first type (without possible connection to the Internet) are not taken into account. Only devices that have an Internet connection, either direct or indirect, will be relevant in an IoT system.

In this work, devices capable of connecting to the Internet directly through TCP/IP protocols are considered as type A devices and those devices that need a Gateway to exchange information through the Internet are type B devices. Therefore, type A devices have a network interface capable of implementing a TCP/IP protocol (typically through an 802.11 WiFi protocol) that allows them to be directly connected to the Internet. Thanks to this connection, it is possible to send and receive data from an external distributed platform. It is therefore understood that these devices are fully integrated in the IoT. On the other hand, type B wireless devices do not have a direct connection to the Internet and need a hardware device that acts as a gateway between them and the network. In this type of device, it is possible to find those wireless sensors that have a connection of the type Bluetooth, Ant +, or Radio Frequency to transmit data. A clear example of Gateway for this type of device is a Smartphone that communicates through one of these wireless protocols to the sensor and transmits this data to the Internet through a WiFi network or a mobile data network.

Depending on the types of wireless devices that exist in the context of the case study in which the system will be deployed, it will be necessary to adopt one solution or another. Next, the two main solutions proposed in this work are detailed.

- For type A wireless devices: The design of a communication middleware capable of connecting the computationally limited device with the multi-agent IoT system through an agent embedded in the device itself is proposed. For this purpose, a communication channel will first be available through a message exchange protocol in which the device will send and receive the middleware information. Once the data has been obtained, the middleware will normalize the information and send it to the reactive agent based on rules in charge of representing the external device within the agent organization. This agent will communicate within the organization with the other agents, who will be responsible for managing the information obtained.
- For wireless devices type B: Not having a direct communication channel with the Internet, it will be necessary to introduce a Gateway device. For the system proposed in this paper, the aim is to develop a software agent embedded in an Android Smartphone device that acts as a gateway between these devices and the IoT multi-agent system. This agent embedded in the Smartphone will be responsible for collecting information from external devices and then transmitting the information to the rest of the agents within the MAS organization.

**Figure 3:**
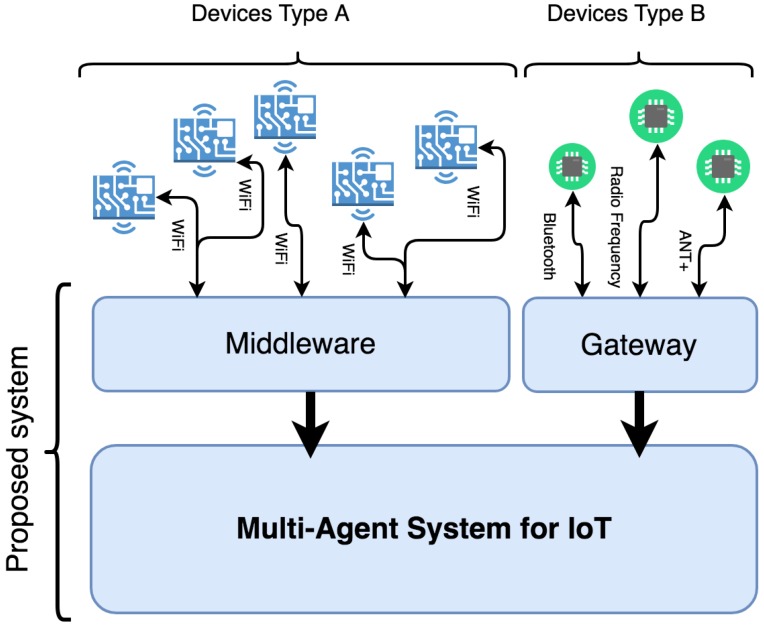
General diagram of the proposed system.

The core part of the proposed architecture, as can be seen in Figure 3, is the multi-agent IoT system. This system is based on a semi-closed organization capable of including new external agents in a controlled manner, guaranteeing stability, confidence, flexibility, and openness. Agents embedded in the devices are presented in the organization through an agent that acts as their representative. Within this system, reactive and deliberative agents will coexist as what is defined as a hybrid system. The chosen methodology for the definition of the steps of analysis and system design will be Gaia [28], in combination with the modeling language AUML (Agent UML). A series of roles and services appropriate to the needs of the designed organization are defined. The communication between the different agents will be carried out directly through the use of the standard protocol FIPA-ACL (Agent Communication Language) [29,30]. This protocol allows the development of interoperable communication structures, which agents can run on different platforms with the objective of exchanging information.

The architecture will have a layer of security with the aim of providing a greater degree of stability and confidence. This system will ensure that only agents with a valid token can join the platform, thus allowing the creation of a semi-closed organization that provides the degree of stability and trust sought. The JADE Framework will be used as the basis for the development and implementation [31]. The agents in JADE are defined as a set of Java classes that encapsulate agent properties, different agent behaviors, interactions, states, and data. The interactions of the agents are carried out with the FIPA-ACL interaction protocols, but the use of other protocols is also possible. One of the most important features is that it allows integration through the Internet by supplying a series of services that let the services provided by agents be invoked, through web services.

The following three sub-sections will detail the three main elements that make up the system proposed in this paper, shown in Figure 3.

### 3.4. Middleware for Type A Devices

As detailed in the previous section, these types of devices have a network layer capable of implementing the TCP/IP protocol for direct communication between the wireless device and the Internet. With the aim of embedding software agents on these devices, it is necessary to design a strategy through which the embedded agent can run independently of the underlying hardware. For this, the strategy of the ‘representative agent’ [32] is used in semi-closed artificial societies, where a software agent that is inside the MAS acts as the representative of the device. This agent will be in charge of communicating with the hardware device through a message queue Publish/Subscribe (Pub/Sub). Thanks to this abstraction, the rest of the agents of the organization will interact with the representative agent as if it was the hardware device. 

More specifically, MQTT (Message Queue Telemetry Transport) [33] has been selected as a Pub/Sub message transport protocol. It is an extremely light protocol and designed for IoT communications, capable of establishing encrypted communications, which gives the network an extra layer of security. The main elements present in the MQTT protocol are the following:

- Client: This is the device responsible for posting messages, subscribing to receive messages, or both;
- Broker: It is the server in charge of receiving the messages published by the clients and then sending them to the rest of the subscribers;
- Publish: When a client sends a message to the broker;
- Topic: Messages must be tagged with a topic. Clients subscribe to a specific topic, and in this way, they only receive the messages published on that topic.

Next, the stages of configuration and operation of the middleware for the type A devices are detailed.

#### 3.4.1. Pre-Configuration of the Devices

Before being incorporated into the IoT system, the devices must complete a minimum configuration to be able to communicate with the rest of the devices and with the server. It is necessary to configure the IP address of the main server on which the devices will have to operate. After this initial configuration, the devices will execute their connection protocol to the server, as is indicated later in this chapter. This is the first and fundamental step in the configuration of the devices.

#### 3.4.2. Registration of the Device in the System

**Figure 4:**
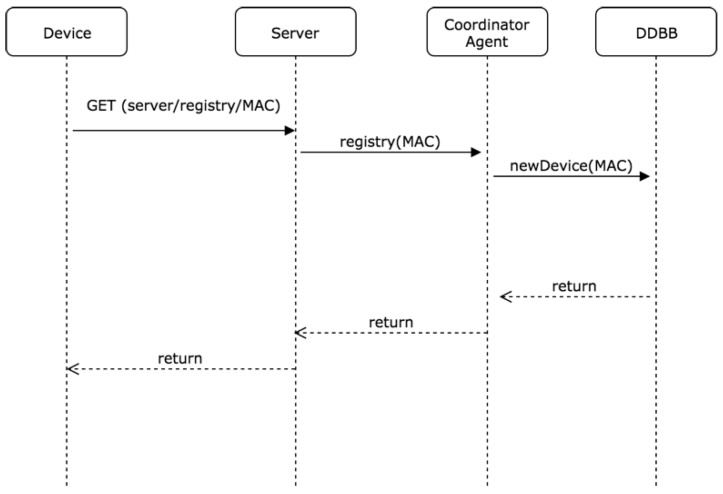
Sequence of registration of a device in the system.

Once the IP address of the server is configured, the device is registered in the system. To do this, the device must make a GET request on one of the endpoints exposed by the API-REST deployed in the system. As shown in Figure 4, the device sends its MAC address, which will be used as an element of univocal identification of the device. After processing the request, the server registers the device in the database and assigns a topic in the server’s MQTT broker. This topic will be the communication channel through which the messages will be exchanged between the server and the device and will be governed by the representative agent of the MAS designed. 

The topic assigned to the device is generated by the server in a random and univocal way, with the aim of adding a higher level of security to the system. The communications through MQTT are encrypted through SSL/TLS, which provides another degree of security, avoiding the injection of packets or malicious data into the system. Thanks to the power of current devices such as ESP8266 or ESP32, it is possible to apply this layer of security where, before, due to the computational cost, it was impossible.

#### 3.4.3. Notification of Device Services

After having registered in the system and having obtained the communication topic with the MQTT broker, the device notifies the system of the services and the resources available to it. This notification is made through an MQTT message where these services are registered so the server has a record of the available services and can then make requests. 

The services and resources available from the device will depend on the environment and the elements deployed in each of the IoT scenarios where the system is implemented. Therefore, these services are well-known by both the servers and the devices before the system is started. A simple example is the device responsible for measuring the temperature and humidity in a room. Table 2 shows a list of services that would implement these devices.

### 3.5. Gateway for Type B Devices

Type B devices, as already mentioned, are those that do not have a direct communication channel with the Internet through which to transmit the measured data. These devices are very numerous in current IoT environments, from heart rate monitors, cadence sensors, and temperature sensors to RFID identification cards. For the system proposed in this work, a mobile agent is implemented in an Android Smartphone device that will act as a gateway between these devices and the distributed multi-agent system.

Currently, Smartphone devices have different communication protocols of a short and medium range, such as Bluetooth, NFC, Ant+, or WIFI. Thanks to these protocols, the devices are able to connect with a large number of type B devices, extending the range of action of the IoT. Smartphones are becoming more common among citizens and are often already fundamental elements for people who live or work in cities. Therefore, these smart devices have become the best candidates to act as a gateway between type B devices and the Internet.

Specifically, thanks to the fact that the JADE platform allows the integration of mobile agents, a mobile agent will be deployed in an Android device, which will be in charge of receiving information from the devices, and will be communicated in the agent with the role of a Mobile Agent of the main IoT multi-agent organization. As detailed in the previous section, mobile agents can perform migration actions when circumstances require it. The following are the three main states in which the mobile agent Gateway of the proposed system will be found.

#### 3.5.1. Mobile Agent as Gateway

**Figure 5:**
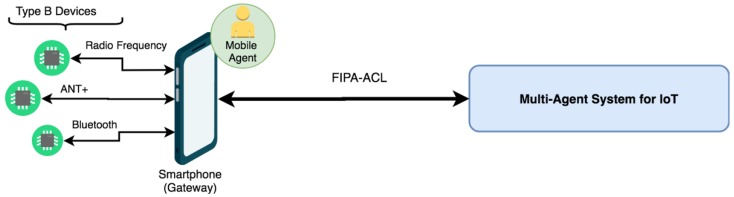
Communication between the mobile agent and IoT architecture.

For cases in which the mobile agent does not require a process migration, this agent will act as a link between the wireless devices of type B, and the main architecture. As shown in Figure 5, it will be responsible for reading the values of the different sensors through the specific protocol that each one implements and, subsequently, it will encode the information with the FIPA-ACL standard to send the data to the architecture. In the same way, the data that arrives from the organization will be sent through FIPA-ACL messages, and this agent will be in charge of communicating with the devices and transmitting the information. It will also have a database to store the different data transmitted in order to avoid a loss of information due to a loss of signal.

#### 3.5.2. Mobile Agent Cloning

**Figure 6:**
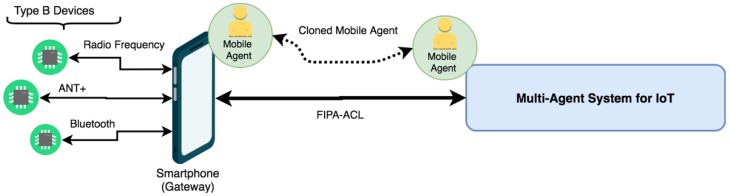
Mobile agent cloning.

In cases in which the system determines that the mobile agent must be cloned, as shown in Figure 6, an exact copy of the agent’s code and status will be made in the main multi-agent system. This cloning may be motivated by an error in the user’s Smartphone, a lack of computational resources (memory or processing), or a low battery level. When the agent is cloned, communication with the wireless devices is not lost as long as the original agent does not cease to be operative on the Smartphone. The main objective is to keep an exact copy of the resources, code, and states of the agent that can serve as a backup once the mobile agent in the Smartphone device has all the necessary resources.

#### 3.5.3. Migration of Mobile Agent

**Figure 7:**
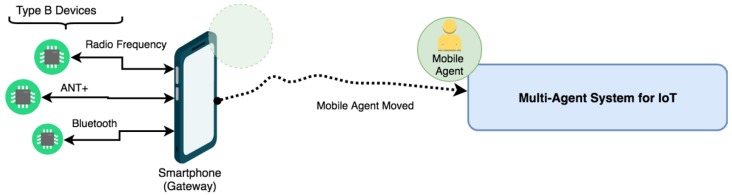
Migration of mobile agent.

For cases in which the original mobile agent ceases to be active for any reason, including those indicated in the previous section, and access to the wireless devices is also interrupted, the mobile agent will perform a complete migration to the main architecture, as is shown in Figure 7. In this way, despite not having communication with the devices, the active data and processes will not be lost and may continue their execution. Once the original mobile agent is recovered, migration to the Smartphone will again occur to continue the normal execution of the agent. This mechanism provides a high level of tolerance to failures in the system. If the integrity of the original mobile agent is compromised, the necessary mechanisms are activated to avoid the total or partial loss of the code and data of the agent and are thus able to resume the immediate execution if it affects the processing of the system.

### 3.6. Multi-Agent Architecture for IoT

This section details the structure and functionality of the multi-agent architecture that will be the core part of the proposal made in this work. As already mentioned in previous sections, the architecture is especially focused on providing services to the IoT through the management of the computationally limited devices that comprise it.

#### 3.6.1. Description of the Architecture

When defining the multi-agent architecture presented in this paper, it is necessary to define a set of fundamental characteristics and properties. The authors in [34] present a series of characteristics that a society of intelligent agents must have:

- A set of agents;
- A set of restrictions within society;
- A communication language;
- A series of roles that agents must play;
- A set of owners or coordinators for the agents.

In the case of the last of the characteristics described, the owner or coordinator of the agents refers to the person or organization that has the power to decide which agents can enter society, what roles they should have, what communication language they should have, and the set of restrictions they will have within society [35]. However, not all companies have an owner in charge of making these decisions and it will vary depending on the design and purpose of the organization. Depending on its purpose, an artificial society of agents needs to apply the following basic characteristics:

- Opening: It is necessary that new agents have the possibility of joining society;
- Flexibility: That refers to the degree of freedom to move that agents have within society;
- Stability: Agents need to anticipate the consequences of their actions;
- Confidence: It refers to the level of trust that the owners or coordinators have in the actions carried out by their agents.

Based on these characteristics, works such as [36,37] perform a classification of the basic types of agent societies: open societies and closed societies.

Within the societies of open agents, it is possible to incorporate one or more agents from the outside into society without any restriction. An agent can join the agent society simply by beginning to interact with the other existing agents. With respect to the four basic characteristics mentioned above, an open agent society has a great openness and great flexibility, but it is highly difficult to ensure that an open society has adequate stability and trust.

On the contrary, in a closed society, it is not possible to incorporate new external agents into the organization. The agents are previously programmed by the system designer and therefore it is known at all times what agents there are, how they interact with each other, and what roles they can play. According to the four basic characteristics explained before, this type of company provides great stability and trust, but lacks an acceptable openness and flexibility. The majority of the multi-agent systems developed at present are systems built on the basis of closed societies. 

Due to this lack of alternatives when designing agent organizations, the authors of [32] propose in their work the incorporation of two new types of societies, with the aim of providing greater diversity. Specifically, the authors describe these two new companies as: semi-open companies and semi-closed companies. 

**Figure 8:**
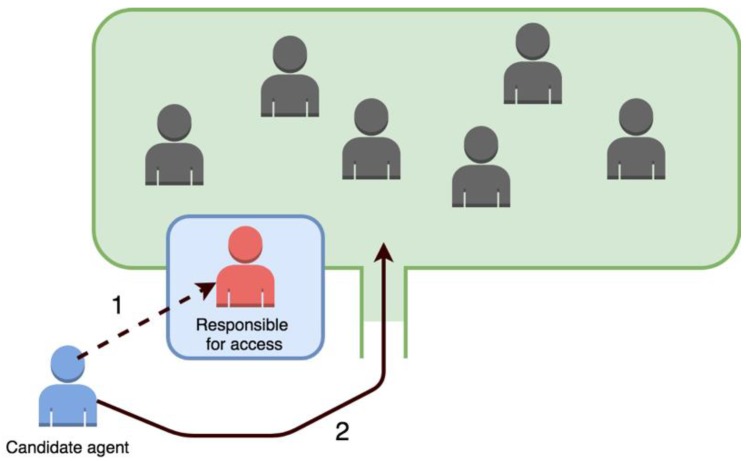
Access of an external agent in a semi-closed society.

In semi-open societies, the figure of the person in charge of access is implemented, who will be in charge of determining if an agent can join the organization or not. As shown in Figure 8, first, the agent seeking to join the organization requests access to the person in charge, and once it is determined that the agent is suitable for incorporation (or not), this proceeds to access to the rest of the agents. The person in charge of access must evaluate if the candidate agent is trustworthy and, therefore, able to access. It is also possible to implement different levels of access, depending on the level of trust assigned by the person responsible for access. 

**Figure 9:**
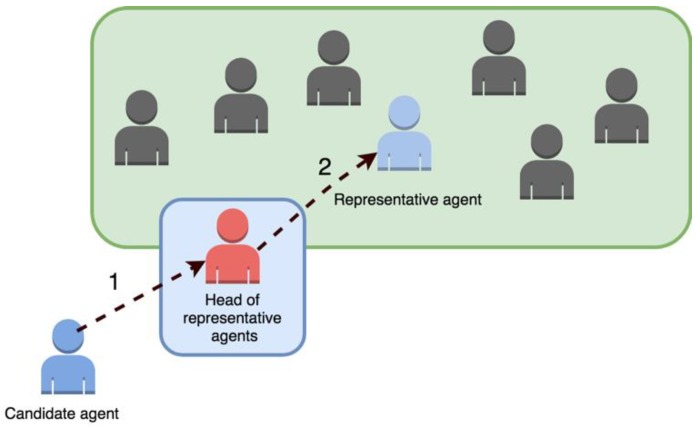
Creation of a representative agent in a semi-closed society.

On the other hand, in semi-closed societies, external agents are not allowed to enter the organization. However, they have the possibility of starting a new agent within the society that will act on behalf of the external agent, as shown in Figure 9. This operation is carried out through a representative agent responsible for whom the candidate agent contacts and requests access to. The representative agent created will be an agent previously pre-defined by the system designer. These types of companies have a greater potential when it comes to implementing good stability and trust, while providing adequate flexibility and openness for the inclusion of new external elements. In this type of company, it is very clearly delimited what optimal access flexibility will produce an open system, but at the same time one which is secure and under the control of the organization administrator. Maintaining control over the architecture is a prerequisite to apply many of the multi-agent coordination paradigms [38]. 

As previously mentioned, the agent society implemented in the architecture proposed in this paper follows the principles of a semi-closed society. Each of the external hardware devices that make up the set of IoT devices has an external embedded agent that has its representative in the MAS architecture. 

**Figure 10:**
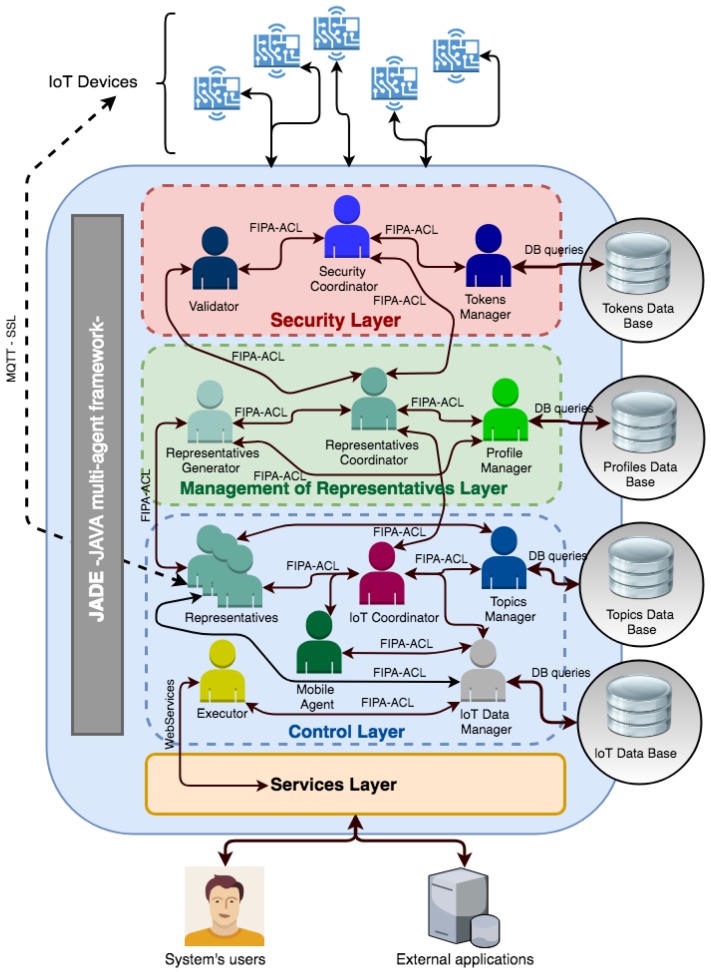
General scheme for the proposed architecture.

As shown in Figure 10 and described in previous sections, the architecture is built on the JADE framework as a basis for its implementation. The first sub-organization is identified with the security layer implemented in the system, with the aim of filtering all communications that are made from outside the organization. To ensure that external agents are really who they claim to be, in this layer, a validation of the tokens associated with the communications made is performed. For this purpose, there is a database where all the valid tokens of the system will be stored and registered. The agent management layer is built on the sub-organization responsible for the management of representative agents for agents embedded in hardware devices. In this layer, there is a database where all the possible profiles of the newly created representative agents are registered. 

The control layer is the main sub-organization of the architecture and will be in charge of managing both the communications of the devices, as well as all the information sent by the devices and the management and execution of the tasks required by the users of the system.

The agents that compose this architecture follow the BDI deliberative model [39,40], in which an agent possesses a type of mental attitude about their beliefs (Beliefs), desires (Desires), and intentions (Intentions), which represent the states of information, motivation, and decision of the agent, respectively. Therefore, as BDI deliberative agents, they can make use of reasoning mechanisms and learning techniques to perform management functionalities and coordination of them according to the particularities of the context in which they are executed.

#### 3.6.2. Security Layer

This sub-organization will be responsible for managing the security of the architecture, analyzing the communications that arrive from the outside, and validating that the access tokens are valid and active in the system. The three roles involved in this layer that are analyzed below are: Security coordinator, token manager, and validator.

Security Coordinator Role: The Security Coordinator role is primarily responsible for the security and trust of the system in its relationship with agents external to the organization. The agent in charge of this role is the first to receive requests for access by external elements, so it must convert the MQTT messages to the FIPA-ACL standard. It must be verified that the sender of the message comes from a reliable source and that the structure of the sent message is correct and does not contain errors or faults. In the event that the external agent has a token, the agent with this role sends it to the agent with the Token Manager role, which proceeds to check it in the token database enabled in the system. In the event that the token is correct, a message is issued to the agent with the Validator role to proceed with the request of the external agent. This role is also in charge of connection requests through the REST web service for the assignment of a topic MQTT.

Token manager role: As mentioned in the previous section, the agent with the Token Manager role is responsible for checking if the token is valid and is registered in the database. In the same way, this agent is in charge of notifying the agent with the coordinating role of Security if the token is valid or if it is not. The behavior of this agent based on that indicated follows the reactive model. Therefore, rules are defined so that the agent, after checking the token in the database, determines if it is valid or not.

Validation role: The agent with the validating role is responsible for processing access requests after verifying that the tokens are correct, or that it is a request from a new external agent. This agent will be in communication with the coordinator of the representative agents and will be in charge of deciding if it is necessary to start the process of creating a new representative agent.

#### 3.6.3. Management Layer of Representatives

This layer is related to the sub-organization responsible for managing the requests of external agents for the creation of new agents that act on their behalf within the organization. The existing roles that are detailed below are: Representative Coordinator, Profile Manager, and Representative Generator.

Coordinator of Representatives: The agent with the role of Coordinator of representatives will be responsible for managing the incorporation of new agents representatives within the organization. First, it must determine if the external agent that has requested the creation of a representative is suitable for its inclusion, if it complies with the security requirements, and if it implements the necessary services for the profile it requests. In the case of accepting the request, this agent issues a message to the agent with a Profile Manager role to start the creation process. In the event that the request refers to a representative agent already created and existing in the organization, this coordinator forwards the original message to the IoT coordinator of the control sub-organization.

Profile Manager Role: Once the agent with the role Coordinator of Representatives has given its approval to the creation of a new agent, the agent with role Manager of Profiles is in charge of looking in the database of profiles for the one that best fits the characteristics of the external agent. These profiles are previously programmed according to the case study and deployment environment of the IoT system. After selecting the profile, this agent issues a message to the agent with the Generator of Representatives role, who will ultimately be responsible for its creation.

Generator of Representatives Role: The final person in charge of generating a new agent within the organization will be the agent with the Generator of Representatives role, who will communicate, as previously mentioned, with the Profile Manager agent. Once the new representative agent with the selected profile has been generated, this agent is responsible for registering it in the organization and registering its data so, from that moment, the rest of the agents involved know of its existence.

#### 3.6.4. Control Layer

The control layer will be the main sub-organization of the multi-agent architecture focused on managing the IoT environments in which it will be deployed. The roles involved that are detailed below are: IoT Coordinator, Topics Manager, IoT Data Manager, Executor, Mobile Agent, and Representative.

IoT Coordinator Role: The agent with the coordinating role will be in charge of supervising the correct functioning of the active agents in the system. Through the monitoring of the data sent by the agents, it will be in charge of processing the information and determining if it is necessary for the representative and mobile agents to take any action in the IoT environment.

Role Manager of Topics: The role Manager of Topics is in charge of monitoring the communications that are made through this protocol with external agents. They will be in charge of assigning new communication topics between a representative agent and their external agent. These topics will be stored in a database that will be implemented for this purpose and will be managed by the agent in charge of this role. When a new external agent requests the creation of a new representative agent, the agent with the role Manager of Topics will generate a new topic in a random and univocal way in the system for safe and consistent communication.

IoT Data Manager Role: All the data measured and analyzed by the external hardware devices that are sent to the IoT architecture are analyzed, normalized, and stored by the agent with the IoT Data Gesture role. This agent is in charge of managing the database where all the data obtained is stored. The functionality of this agent is independent of the underlying technology used for the implementation of the database that may vary, depending on the needs and characteristics of the data analyzed in the environment. The agent in charge of this role will also be the intermediary of the agent with the executing role, responsible for sending the requested data in the service layer.

Executing role: One of the main functionalities of the system is to provide services and data to users and external applications that wish to make use of this system. For this, the agent with the Executor role will be the link between the data and the services provided by the system through the service layer. This agent will process the requests that arrive from the service layer and will communicate with the agent with the IoT Data Manager role for the data query. This agent will also check the level of permissions and privileges of the users and applications that request data and determine if the requested data can be accessed by them. The implementation of the agent in charge of this role will depend on the specific case study in which the system is deployed.

Mobile Agent Role: As detailed in previous sections, the so-called type B devices (they do not have a direct communication channel with the Internet) need a communication gateway with the Internet. The agent with the Mobile role will be the representative of the agent embedded in the Smartphone device in charge of communicating with Type B devices. As it is a type of agent capable of migrating its code and status from the Smartphone to the main IoT architecture, it is necessary that the agent with the role Mobile Agent is representative for cases in which the agent is cloned or moved from its original location. In addition, this agent will be responsible for communicating and exchanging data with the original agent from the Smartphone.

Representative Role: The representative role is in charge of representing the external agents that request to enter the organization and are authorized for them. In the case of this system, the external agents are the agents embedded in the limited hardware devices and the component the IoT system implemented. The operation of these agents is key to the correct communication of hardware devices with the rest of the system. They are in charge of direct communications through MQTT with external agents and are responsible for transforming the messages that arrive through JSON messages into standardized FIPA-ACL messages so that they can be delivered and read by the rest of the agents of the organization.

The possible profiles that these agents will have will depend on the cases and environments in which the system is deployed. It will be necessary to differentiate three types of general profiles from which the rest of the profiles will be derived: sensor profile, effector profile, and mixed profile. In cases in which the hardware devices have one or more sensors and send the measured data to the organization, a representative agent with a sensor profile must be generated. In those cases in which the device is equipped with only one or several effectors (actuators), a new representative agent with an effector role must be generated. For cases in which one or more sensors are available in combination with one or several effectors, the profile of the representative agent generated will be mixed.

#### 3.6.5. Services Layer

This layer includes the services and functionalities offered by the IoT architecture on the data and devices active in the system. It will be possible to access these services in a ubiquitous and distributed way so that the computer systems that support the multi-agent architecture are not affected. The services and functionalities to be implemented will depend on each study and environment in which the system is deployed. The objective of this research work is the analysis and design of the upper layers previously described, and the final implementation of this layer is not focused. It will be possible to apply proven strategies with good results in other research works such as [41,42], where they apply subscription systems to define the services and functionalities that will be accessed by users and applications of the system.

### 3.7. Security and Trust

Now the structure and functionality of the multi-agent architecture has been described, which will form the central part of the proposed system, this section analyzes the security and trust mechanisms in the system. In particular, when addressing security, as previously introduced, the system implements encryption over SSL/TLS to achieve a strong encryption of messages. Both ESP8266 and ESP32 devices have the necessary capacity to execute these encryption protocols without affecting their performance. Specifically, the ESP8266 can work as an SSL client or as an SSL server. Thanks to this native functionality, it is possible to generate bidirectional authentication with the server, in such a way that the messages exchanged between the embedded agent and the rest of the organization are protected and encrypted. The agent with the role Security Coordinator will be responsible for managing the network of certificates between agents.

**Figure 11:**
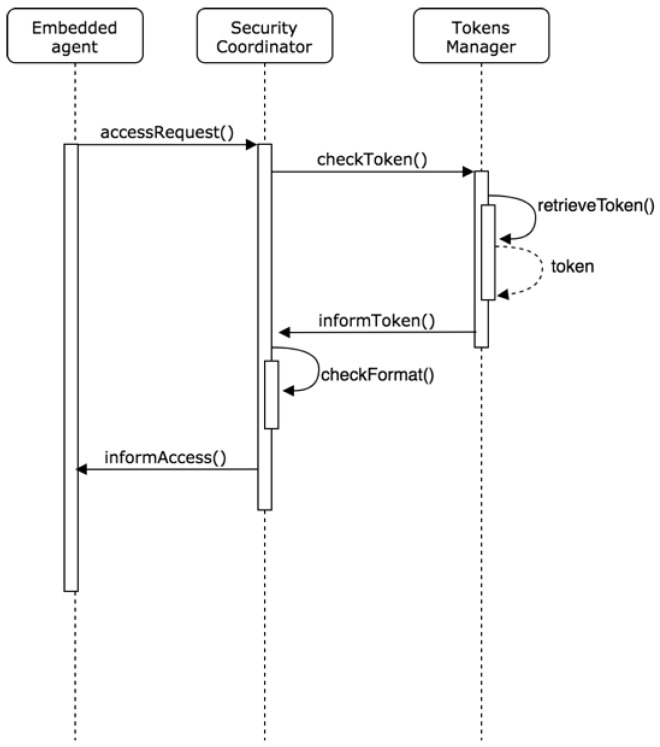
Relationship between the embedded agent and the security coordinator.

When it comes to providing a greater degree of confidence and avoiding anomalous behavior in embedded agents, the figure of the representative agent has been implemented. As mentioned in the previous section, thanks to the implementation of the representative agent mechanism, all the new instances that correspond to a new embedded agent will be created from within the organization. Being created from within, the system ensures that the behavior of the agent is clearly defined and strictly limited to the functions assigned by default. Below are some of the sequence schemes involved in the security and trust of the system. Figure 11 shows the relationship between the embedded agent and the security coordinator when processing an external access request with a previous token. In this relation, the token manager checks the validity of the sent token.

**Figure 12:**
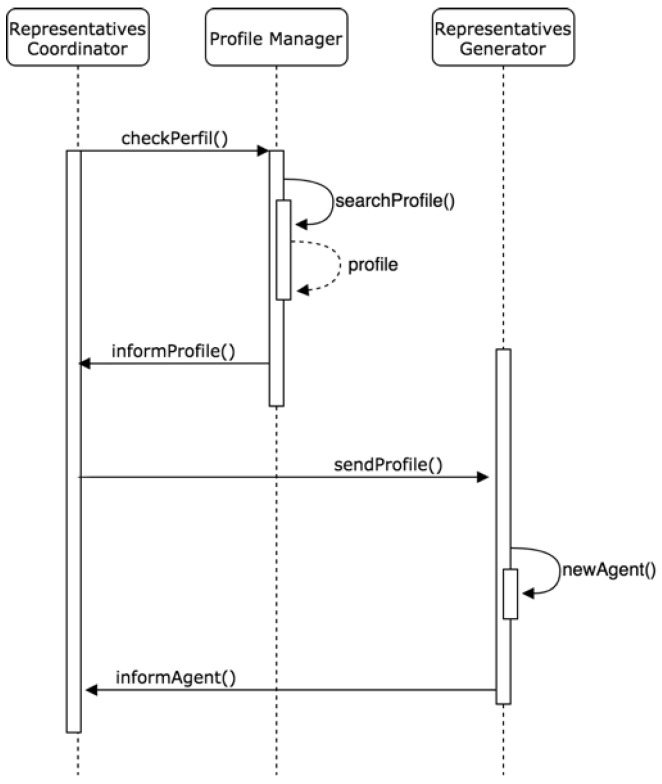
Process of consulting the profile of a new agent.

The diagram of Figure 12 shows the relationship between the three agents of the sub-organization for the management of representative agents. Specifically, the process of consulting and sending the profile that the new representative agent generated will have is shown. In this way, agents outside the organization are prevented from injecting malicious external codes into the organization. All the agents included in the system are registered by the system itself.

**Figure 13:**
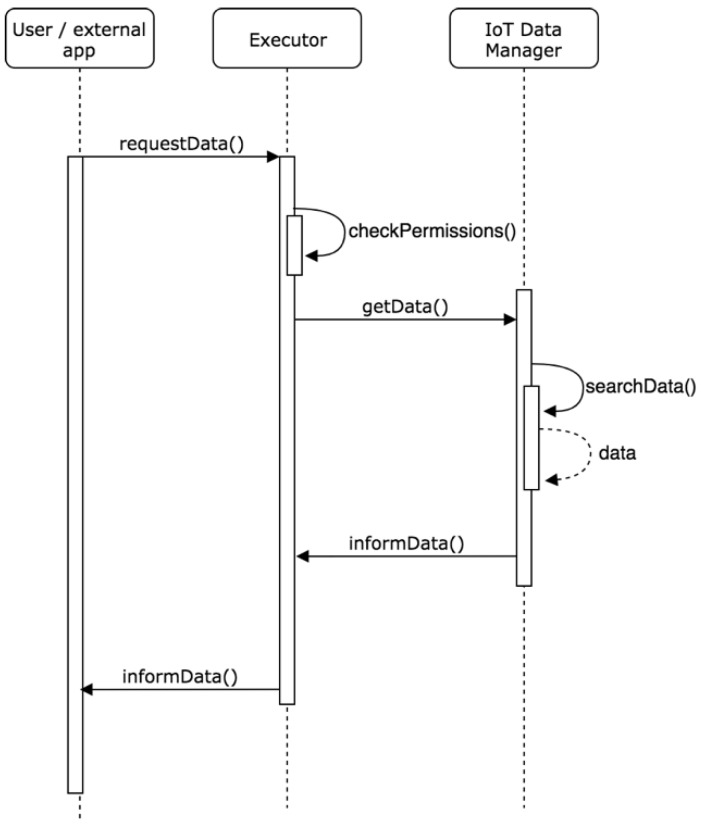
Process of information exchange from outside the system.

The diagram in Figure 13 shows the interaction between a user or external application and the agent in charge of processing and executing requests for access to data that arrive to the system, once it has been verified that the permissions are correct. In this way, security and confidence are assured when sending the data generated by the system. 

## 4. Case Study

After having analyzed the current state of the art and having detailed the proposed system in this work, the system designed in a real IoT environment will now be put into practice. Through the implantation of different sensors on a bicycle, it is possible to obtain interesting data about the route taken, the effort of the user, and the behavior of the vehicle. The transport sector is one of the most important fields of action in the IoT. Therefore, building intelligent environments that seek to improve the transport and mobility of vehicles in cities and roads, while optimizing energy and safety resources, is essential when it comes to achieving sustainability in the cities of the future.

Then, in the following sections, the case study will be carried out in a real environment, based on the system proposed in this work, where it will monitor the routes made by an electric bicycle. The registration of these data may be used in future work in areas such as safety, energy optimization, or the comfort of bicycle users. It is a simple case study whose main objective is to illustrate and validate the functioning of the system proposed in this work. More specifically, the case study will be carried out through an electric bicycle since it has been a very popular type of vehicle in recent years and provides a series of sensors and extra safety elements that improve the efficiency of the system. In any case, the system can be implemented in the same way in a conventional bicycle through the implantation of the necessary sensors. In order to measure the efficiency of the proposed system, this system has been compared with a traditional system based on sending data. Three fundamental aspects, including battery life, processing time, and ability to incorporate new nodes, are compared. 

### 4.1. Architecture Implementation

**Figure 14:**
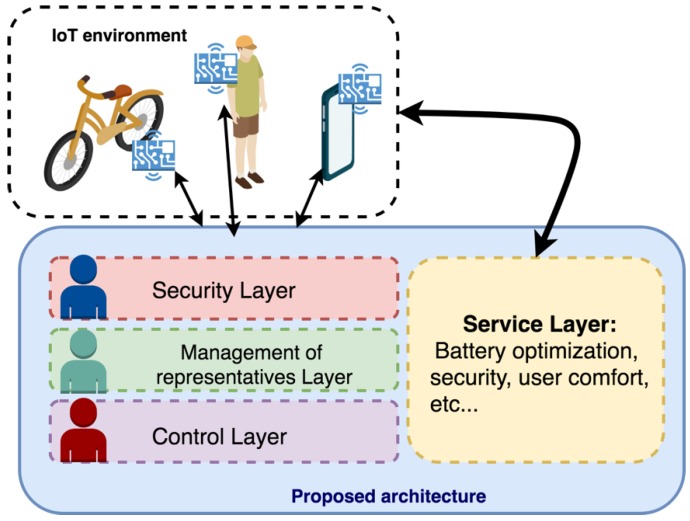
General scheme of the proposed system.

This architecture is composed of a sensor network integrated into the bicycle, as well as a sensor network deployed in the user’s Smartphone device and on the user’s own body. Thanks to them, it is possible to analyze all the data that occurs during the realization of a bicycle tour. Some of these data are: Heart rate, battery level, or wheel speed. Figure 14 shows the general scheme for this proposed system in the case study. As can be seen, within the proposed architecture are the three sub-organizations described above and that correspond to the multi-agent system proposed in this paper. 

The sensor network deployed in this case study that makes up the IoT environment is the most commonly used for electric bicycles and practically all commercial electric bicycles already have it. When a user travels a route on an electric bicycle, it is important that certain parameters are recorded during the trip to provide useful information, such as speed, battery consumption, GPS, and fundamentally, the Smartphone’s accelerometer.

### 4.2. Sensor System

**Figure 15:**
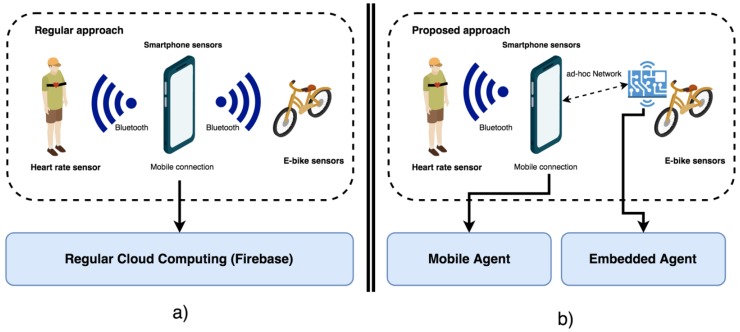
(**a**) Regular approach system based on cloud computing service. (**b**) Proposed approach based on Mobile Agent and Embedded Agent.

Figure 15b shows the deployed sensor system that will act as the IoT layer of the proposed architecture in the case study. The user’s Smartphone plays a double role: on the one hand, it makes use of different sensors that have the vast majority of devices, such as the GPS sensor, compass, or accelerometer; on the other hand, it provides a mobile data network to the ESP8266. Connected to this device and through a Bluetooth connection are the rest of the elements of the system. One of them is the electric bicycle, which provides basic information, such as speed, energy consumption, or battery charge. On the other hand, it is possible to connect different external sensors, such as the heart rate monitor responsible for measuring the heart rate of the user during the performance of an electric bike tour. Figure 15a shows a classic approach to the problem through the use of cloud computing services such as Firebase. This regular approach aims to compare the efficiency of the architecture proposed in this work. 

**Figure 16:**
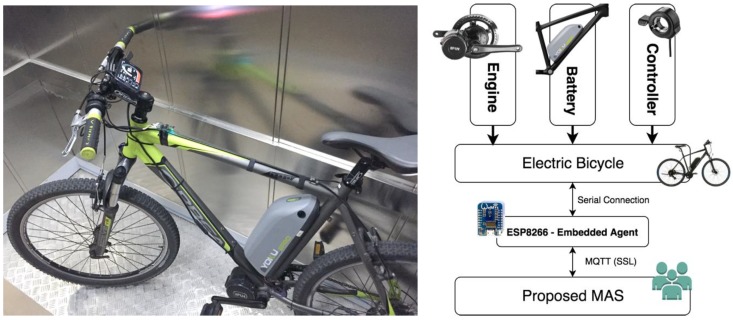
Sensorization system deployed on an electric bicycle.

Specifically, in this case study, an electric bicycle of the Orbea brand has been used, to which an IoT node has been added through an ESP8266, as can be seen in Figure 16. In this way, all the data that are measured by the system are managed by the IoT node on which the embedded agent is deployed that will act as an external agent in the MAS designed architecture.

### 4.3. Results

**Figure 17:**
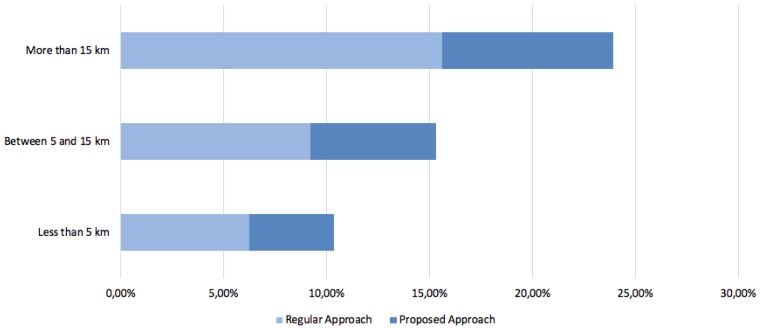
Comparison of average battery consumption.

As previously mentioned, the case study carried out in this work compares a classical approach based on a sensorization and cloud processing system with a new approach based on embedded intelligent agents. To carry out this comparison, the data collection platform for electric bicycles ebikemotion developed in collaboration with the research group of the University of Salamanca has been used. This platform has more than 5000 active users around the world who send data on their electric bike routes daily. Thanks to the data collected through the traditional approach, it is possible to compare the data of the routes obtained by the users who have tried the system proposed in this work. First, it is possible to compare the results on battery consumption by the system. Thanks to the continuous sending of the battery charge, it is possible to measure the efficiency of the system. The data have been categorized based on the distance traveled by the users on each route, obtaining three groups of routes: Routes of less than 5 km, routes between 5 and 15 km, and routes of more than 15 km. As shown in Figure 17, the battery consumption (battery of the electric bicycle plus Smartphone battery) is always lower in the routes where the proposed system has been used. This reduction in battery consumption is mainly due to the fact that the information in the regular system is processed in its entirety in the mobile device, and as a continuous communication of input/output of data within the cloud environment; while in the proposed system, the computing of the data is distributed in the different IoT devices deployed. Likewise, communication with the agent architecture involves more agile and lighter communication in the sending of data than that produced with regular cloud environments.

**Figure 18:**
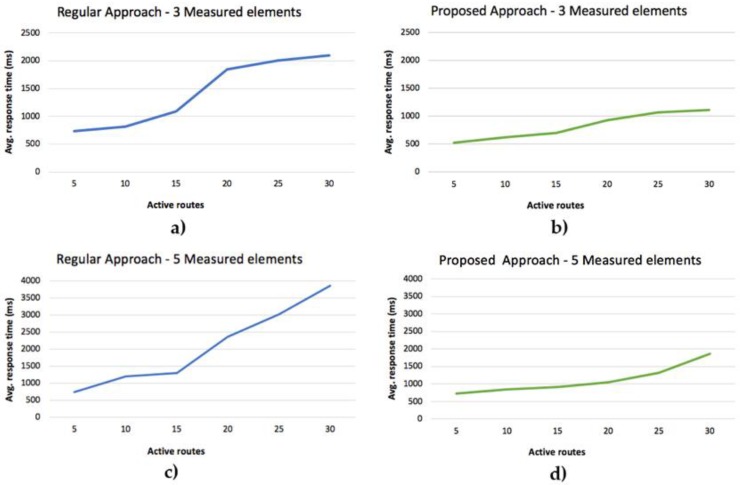
(**a**) Regular approach and three measured elements. (**b**) Proposed approach and three measured elements. (**c**) Regular approach and five measured elements. (**d**) Proposed approach and five measured elements.

Other data comparatively analyzed in the study was the processing speed of the different data from the electric bicycle. It should be noted that in the traditional system, the data is sent in a raw format to the server that later is responsible for filtering and pre-processing the data, correcting GPS locations, and adjusting the altitudes of the route. In the case of the proposed system, the embedded agent is in charge of the previous data filtering (avoid duplicate data, null values, etc.) based on the characteristics sent from the representative agent of the IoT device. Despite this preprocessing task, the speed at which the system stores the final data filtered from the route is more optimal than in the traditional approach, as can be seen in Figure 18.

The routes that only have three measurement values (speed, assistance level, and battery) have been compared to those with five variables (speed, attendance level, battery, heart rate, and cadence). After analyzing the average response time of both proposals, it can be observed that the proposed system provides a significant improvement in the response times. It is also possible to observe how the increase of users (active routes) does not greatly affect the execution times, which remain practically constant, unlike the classic approach.

**Figure 19:**
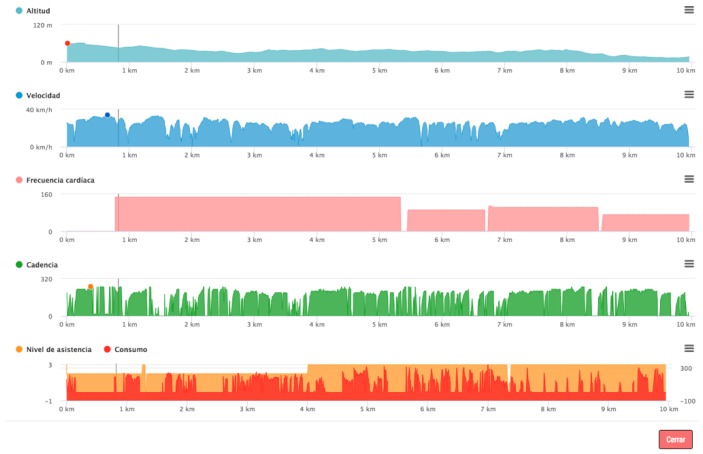
Data collected by the sensorization system on a bicycle route.

As a final result of the case study, an open and scalable system has been designed, which is capable of obtaining all the data produced while performing an activity on an electric bicycle in real time. It is a system capable of incorporating new sensors to add new accurate information or to replace the sensors with different ones, without the need to reprogram the system. In Figure 19, the data obtained by a bicycle after being stored in the central server, where it will later be analyzed in search of possible optimizations or on which real-time monitoring can be performed in search of security elements such as falls, accidents, or abnormal driving behavior, can be viewed.

## 5. Conclusions

In this paper, an intelligent system based on agents embedded in wireless devices with reduced capacities (memory and limited computing capacity) for Internet of Things (IoT) environments has been presented. 

This system is integrated into a multi-agent architecture that will be responsible for managing the different wireless devices that make up the IoT scenario in which the system is deployed. Two main types of wireless devices that can be deployed have been identified: Wireless devices capable of exchanging information with the Internet and devices that require a gateway that acts as a link between the device and the Internet. These two types of devices have been called type A devices and type B devices, respectively. 

For each of them, a different strategy is proposed when embedding software agents on them. For type A devices, a communication middleware based on MQTT and a semi-closed agent architecture has been designed, where a representative agent acting on behalf of the device is generated within the MAS architecture. For cases in which the devices do not have a direct communication channel with the Internet (type B), it is necessary to introduce a Gateway device. In the system designed in this work, a mobile agent is introduced in a Smartphone device that acts as a gateway between these devices and the multi-agent system. This mobile agent is responsible for collecting information from external devices and then transmitting the information to an agent within the organization. 

Regarding the IoT multi-agent organization designed in this work, it is worth noting that it is a semi-closed organization able to include new external agents in a controlled manner, guaranteeing stability, trust, flexibility, and openness. For this, the MAS system is organized into four different layers. The first is a security layer where all the messages that arrive from the outside of the system will be filtered, avoiding the malicious injection of messages or the communication with previously unregistered agents. A second layer will be in charge of the management of new representative agents that act on behalf of the wireless devices. The third layer contains all the agents responsible for IoT data management and the coordination of the architecture. The service layer is open and may contain mechanisms and techniques for processing and analyzing different data, depending on the environment in which the system is deployed.

Finally, in order to validate the proposed system, a case study was carried out in a real IoT environment. In the same way that the system proposed in this paper has been successfully deployed in an IoT environment, such as a connected electric bicycle, it is possible to deploy this system in other environments. The progressive success experienced by the IoT phenomenon and the connected cities, raises an endless number of possible scenarios where the integration of the proposed system in this work can mark an advance with respect to other systems proposed in the current literature.

As future work, it will be possible to analyze alternatives in the security layer to include new paradigms of security and trust that improve the robustness of the system. It will also be a future line of research to implement the proposed system in massive environments where sensor systems include thousands of devices. This will allow, among other things, to check the flexibility when adding large sensor networks with important amounts of software agents embedded in the system. Another point to be addressed in future research is the combination of techniques for data monitoring, sampling, and filtering to obtain an improvement in the performance of IoT devices. As has been demonstrated in works such as [43,44] or more recently in [45], implementing adaptive algorithms of sampling and filtering for the data generated in the environment can lead to an even greater optimization in aspects such as the volume of data generated or the battery consumption. This is a very interesting field of research, and the combination of different methodologies and approaches will lead to fully intelligent environments in the near future.

## Figures and Tables

**Table 1:** Main requirements of intelligent environments and smart objects.

Requirement	Domain
Req. 1 (SE)	Abstraction over heterogeneous input and output hardware devices
Req. 2 (SE)	Abstraction over hardware and software interfaces
Req. 3 (SE)	Abstraction over data streams (continuous or discrete data or events) and data types
Req. 4 (SE)	Abstraction over physicality (location, context)
Req. 5 (SE)	Abstraction over the development process
Req. 6 (SO)	Heterogeneity and Application Development
Req. 7 (SO)	Augmentation Variation of Smart Objects
Req. 8 (SO)	Management of Smart Object
Req. 9 (SO)	Evolution of Smart Object Systems

**Table 2:** Example of services available in a device responsible for measuring the temperature and humidity of a room.

Service Name	Input/Output of Data	Description
sendTemperature	Data output	Service used by the device for sending temperature data
sendHumidity	Data output	Service responsible for sending moisture recorded by the sensor
setUnitsTemp	Data input	Service responsible for configuring the units of measurement of temperature (Centigrade or Fahrenheit)
setSampling	Data input	Service responsible for configuring the sampling rate with which sensor readings are collected
